# From the World to Western: A Community-Engaged Teaching Strategy to Enhance Students’ Learning of Cultural Issues Relevant to Healthcare

**DOI:** 10.3390/ijerph19095114

**Published:** 2022-04-22

**Authors:** Olayide Ogunsiji, Anita Eseosa Ogbeide, Valentine Mukuria, Florence Olugbemiro, Alex Workman, Tinashe Dune

**Affiliations:** 1 School of Nursing and Midwifery, Western Sydney University, Locked Bag 1797, Penrith, NSW 2751, Australia; f.o@westernsydney.edu.au; 2 School of Health Sciences, Campbelltown Campus, Western Sydney University, Locked Bag 1797, Penrith, NSW 2751, Australia; a.ogbeide@westernsydney.edu.au (A.E.O.); a.workman@westernsydney.edu.au (A.W.); t.dune@westernsydney.edu.au (T.D.); 3 Office of People and Success, Western Sydney University, Locked Bag 1797, Penrith, NSW 2751, Australia; v.mukuria@westernsydney.edu.au; 4 School of Social Science, Western Sydney University, Locked Bag 1797, Penrith, NSW 2751, Australia; 5 Translational Health Research Institute, Western Sydney University, Locked Bag 1797, Penrith, NSW 2751, Australia; 6 Diabetes Obesity and Metabolism Translational Research Unit, Western Sydney University, Locked Bag 1797, Penrith, NSW 2751, Australia

**Keywords:** cultural immersion, cultural competency, community-engaged learning, CALD groups, cultural facilitators, teaching and learning process, health students

## Abstract

Using the transformational learning theory and action research method, this study captured the experiences of students from health-related disciplines in the cultural immersion program From the World to Western. A total of nine students participated in the pilot program with four host families from Culturally and Linguistically Diverse (CALD) backgrounds, and four cultural facilitators who connected the host families and students. The findings of this research showed that it was beneficial for students in health-related disciplines to engage in the cultural immersion program to further prepare them for culturally competent care in their future roles as healthcare professionals. In addition, the students indicated the need for the cultural immersion program to be part of the curriculum for future students to develop cultural skills, awareness and encounters with diverse populations.

## 1. Introduction

Australia is one of the world’s most ethnically diverse nations, with nearly a quarter of Australian inhabitants born outside of the country [1]. Culturally diverse migrant groups underutilise health services in their new countries, and cultural barriers are cited as reasons for not using health services [2,3,4]. Achieving culturally appropriate healthcare is critical, especially in light of the ever-changing cultural makeup and the existence of numerous ethnic groups within Australian society [5]. Culture is core to an individual’s health and wellbeing, and a defining characteristic for individuals in every locale around the world [6,7]. Therefore, having a deeper understanding of how behaviours are rooted in an individual’s unique cultural experience and as a response to social constraints might better equip health professionals with the context, skills, and empathy necessary for providing holistic treatment [8]. This understanding has the potential for improving the health outcomes of CALD populations, as healthcare providers consider an individual’s life experiences and culture when assessing their health and treating their illnesses [9]. According to Smith et al. [10], all health professionals have a societal obligation to be equipped to recognise and respect cultural differences and beliefs. To meet this challenge, students from health-related disciplines who are future health practitioners must begin to develop the skills necessary to offer competent treatment while maintaining culturally sensitive communication, regardless of cultural differences [11,12]. For this skill development to occur, there is a need to continuously learn cultural competency and safety pedagogies [13].

### 1.1. Cultural Competency

In the field of healthcare, cultural competency is a core pillar that aims to decrease current inequities in the delivery of culturally sensitive and high-quality services [14]. Fundamentally, it attempts to ensure that all patients receive care tailored to their requirements [15] and ensure that all patients have equal access to healthcare across varied groups. According to a comprehensive definition by Bainbridge et al. [16], culturally competent services recognise and respect the diversity amongst patients and the socio-cultural elements that may influence their health, including patients’ beliefs, behaviours, attitudes, and language. As populations become more varied due to globalisation and migration, health professionals are increasingly finding themselves providing care to patients who have a variety of cultural and language requirements [17]. However, numerous studies [18,19] have stated that teaching and learning cultural competency in the classroom is no longer enough, because students in health-related courses do not understand the concept in-depth. For instance, the study of Collins and Pieterse [20] indicates that cultural competence training must incorporate a new degree of infusion, “reaching beyond the classroom walls” (p. 17), and immediately integrating multicultural issues to trainees’ daily lives, underlining the need to integrate classroom information and real-world situations. Among the educational methods developed to increase cultural knowledge and sensitivity in the education of healthcare professionals is “cultural immersion” [5]. Cultural immersion creates opportunities for transformational learning through direct interactions with culturally diverse populations [5], which is effective.

### 1.2. Cultural Immersion

Academics have proposed that students tend to focus on the theoretical components of diversity while neglecting the affective responses that arise when dealing with unfamiliar surroundings, reducing their ability to empathise with a wide range of client populations [21]. As a result, it is critical to provide students with learning opportunities that allow them to directly participate in a learning process in which they examine, expand, and question their cultural assumptions, resulting in affective, behavioural, and cognitive shifts that promote higher levels of awareness [21,22]. In order to build higher cultural responsiveness, cultural immersion programs are an important educational activity to engage in [23]. They require students to go into unfamiliar situations and consider other people’s worldviews and lifestyles, which can be tricky [24,25]. According to Delano-Oriaran [26], cultural immersion field experiences provide an opportunity to further expand one’s understanding of culture’s influence on teaching and learning. As a result, it is critical to provide students with learning opportunities that allow them to directly participate in a learning process [27] in which they examine, expand, and question their cultural assumptions, resulting in affective, behavioural, and cognitive shifts that promote higher levels of awareness [21,22].

According to Burnett et al. [28] and West-Olatunj and Shannonhuse [29], the experience of being ‘other’ can be provided by cultural immersion programs, illustrating the difficulties faced by minority clients/patients while also emphasising the importance of cultural sensitivity in helping professions. This experience can be achieved through experiential involvement and immersion in the social, political, cultural, and environmental realities of CALD communities. This is likely to provide a critical experience for students in health-related disciplines which cannot be attained solely through didactic instruction. This point is buttressed by research of Arredondo and Toporek [30], Coleman [31], and Collins and Pieterse [20], where they individually suggested that there are long-term benefits in engaging students in real-world contexts outside of the classroom through mixing theory and practice. Hence, this project aims to develop a cultural immersion program to prepare students who are passionate about health-related fields and are interested in managing and supporting the health and wellbeing of culturally and linguistically diverse populations and evaluating their experience.

### 1.3. Research Context

This project draws from a previous mixed-method study conducted among second year nursing (*n* = 154) and interprofessional allied health students (*n* = 273) at Western Sydney University (WSU), exploring their experiences of learning about the role of culture in healthcare for nursing students and the development of self-reflection and cultural competence for Allied Health students. This research highlighted the concerns of sampled students regarding their preparedness to support, in a culturally safe way, CALD groups who are accessing healthcare services [32]. Based on this finding, the cultural immersion program pilot experience was birth, which allowed for students from health-related disciplines to safely explore cultural encounters which are not their own.

Students who indicated interest in the immersion program were required to spend a day with a family from another cultural background that is different from theirs. The cultural immersion facilitator (who connected the host families and students) intermittently observed the interaction between the students and the families they were allocated to. This observation was very crucial in determining the level of enthusiasm and willingness to ask and answer questions throughout the duration of the interaction. This observation is used in explaining the quality of the cultural encounter and cultural skills, which are crucial for cultural competency [33].

## 2. Materials and Methods

### 2.1. Theoretical Framework: Transformative Learning Theory

Transformative learning [34,35,36,37] is the process of effecting change in a frame of reference. According to Mezirow [38], as humans we understand our experiences through frames of reference, which are structures of assumptions that we hold about the world. Expectations, perceptions, cognition, and feelings are all selectively shaped and delimited by them. They determine our “course of action”. Once a pattern is established, we automatically go from one specific action (mental or behavioural) to another [38]. Those concepts that do not conform to our expectations are immediately dismissed as aberrations, gibberish, unimportant, odd, or erroneous, and we label them as unworthy of investigation. From evidence provided in the literature review [20,30,31], cultural immersion experience allows students in health-related fields the space to shift their frame of reference to be more inclusive, sensitive, self-reflective and integrative of experience when the circumstances allow it. Transformative learning, as Buttigieg and Calleja [39] explained, necessitates transformative experiences, which are situations that push the individual to confront any current meanings and discover new ones.

### 2.2. Research Design

This study utilises an explorative approach that uses the action research method which feeds the aim of the study, as the cultural immersion program looks to improve skills, practice and proficiency of students in health-related disciplines in cultural competency and safety towards CALD groups. Action research is a practice–improvement strategy [40]. It entails taking action, evaluating it, and critically reflecting on it, after which adjustments in practice are made based on the evidence obtained. Individuals with the same goal conduct action research, which is participatory and collaborative. It is situational and context dependent. It encourages reflection that allows the students of health-related discipline a space to consider their positioning in the production of meaning from the experience. Further, according to Koshy et al. (p. 1) “If the answer to the problem leads to an improvement in practice, action research might include problem solving”. This is buttressed by Meyer [41], who states that action research focuses on developing answers to practical problems (in this case, the challenge of providing culturally competent healthcare) and its capacity to empower practitioners (in this case, students in health-related disciplines).

### 2.3. Sample and Recruitment

The participants in this study include 3 distinctive groups: the students (*n* = 9) from health-related disciplines/programs in WSU, the host families (*n* = 4) from refugee or migrant backgrounds living in the Western Sydney region, and the case officers (*n* = 4) known as the “Cultural Immersion Facilitators” from a migrant resource centre in Western Sydney who have existing relationships with the host families. A convenience sampling technique were used to recruit participants for the study. This technique was suitable for the researchers as samples were conveniently located [42] within the schools of health-related disciplines in WSU campuses and the community of the host family. The recruitment for the different groups of participants varied, with specific inclusion and exclusion criteria (see Table S1).

The recruitment of students began by sending out a generic email to the Directors of Academic Programs (DAPs) of health-related disciplines at WSU informing them of and introducing this project to them. Other content of the email included the importance of their role and involvement in the project, particularly in accessing students who may be interested in participating in this cultural immersion program. In addition to this, the Students Representative Council (SRC) on each of the campuses was contacted to introduce to and inform students who may be interested in participating in the program. Furthermore, fliers were distributed on the boards and common areas around the university that are frequented by students. In total 15 students indicated interest in participating; however, only 9 students participated in the cultural immersion program. The major reason for the withdrawal of six students from the project was the fear of contracting coronavirus from spending a day with a host family due to the ongoing COVID-19 pandemic during the immersion process. As earlier stated in the research context, the host families and the cultural immersion facilitators were approached through community groups, migrant resource centres and non-government organisations that provide services to CALD families. They were informed of the important role that they would need to play in accessing families that meet the inclusion criteria. Overall, 4 families from Sri Lankan Tamil, Afghanistan, Syrian and Iranian cultural background indicated interest and hosted undergraduate students of health-related disciplines from WSU.

### 2.4. Data Collection

The global COVID-19 pandemic had an impact on the way data were collected from students, cultural facilitators and their engagement with this research and the number of students and host family participants recruited for the study. Based on request from the participants, a semi-structured interview schedule was sent by author O.O. asking students to report on their experience with their host families and reflections, while cultural facilitators were to report on their observation of the student’s engagement with host family. Host families also provided feedback on spending a day with students allocated to them. During the immersion day, students were provided with cultural immersion worksheets to guide the students on the questions they can ask to obtain an in-depth understanding of a range of health issues related to their assigned host family. The worksheet contained questions tailored to answer the research objective. The questions included what health means to them, what role their culture/ethnicity/ethnicity/race/social diversity/ability diversity/(etc.) has on their health-related outcomes, as well as facilitators and barriers to optimal health for their family and community. Students’, host families’ and facilitators’ names were redacted, with pseudonyms assigned prior to data analysis.

Further, a semi-structured interview schedule was sent out to students and facilitators after the immersion day with host family. Students’ semi-structured interview schedule contained questions regarding their experiences with the host families, what was learnt in relation to culture and health and additional comments to improve future immersion programs. The facilitators’ semi-structured interview schedule contained questions about the student experience and host family, what the students and host family like best/do not like about the immersion day, and comments to improve future immersion programs. This provided the research team with an in-depth understanding of the impact of the cultural immersion day for both the student and host family, which informed the result and discussion of the study.

### 2.5. Data Analysis

Data from students’ reflection, worksheet, and semi-structured interview schedule were collected, as well as feedback from facilitators which were collated and thematically analysed [43,44]. One student and one facilitator provided recordings on the semi-structured interview schedule and feedback, which were transcribed and thematically analysed using a deductive approach [45]. The audio recordings were transcribed by author F.O. and O.O. and checked for accuracy by author A.E.O. Based on the work of Braun and Clarke [43]. For all the data sources provided, we employed a theme-coding technique.

This method has been widely utilized and proven to be reliable in a variety of fields, including human health studies [46]. It is a six-step process that comprises familiarizing the researcher with the data through producing initial codes, searching for patterns or themes across data, reviewing themes, defining and labelling themes, and writing the report. We used Lincoln and Guba’s [47] “trustworthiness” criterion to promote trustworthiness while limiting threats to validity. We gave detailed and in-depth descriptive data and quoted the participants in the text to meet the criterion for transferability. To meet the criterion of dependability, each transcription was read, reviewed, and coded separately by two of the authors, O.O. and A.E.O, and final interpretations were obtained through consensus among all authors. Emerging and substantive categories within the participants’ statements were defined in relation to the objectives of the analysis. Analysis focused on topical responses and coding for phrase repetition, clear and emotional comments, as well as dialogue markers.

### 2.6. Ethics and Consent

Before engaging in recruitment and data collection, ethics approval was obtained from an Institutional Human Research Ethics Committee [H13793]. Participation is voluntary and all the participants were provided with a Participant Information Sheet before immersion days took place with each family. Several Zoom meetings were held with the students who consented to participate and with cultural facilitators. Emails were sent to the cultural immersion facilitators to introduce the students who were visiting the host families.

## 3. Results

The results presented here have captured the experience of cultural immersion program through the lens of the students, host family and facilitators. The impact of the cultural immersion experience on students’ understanding and preparedness for culturally competent care for persons of CALD backgrounds was also captured. The key themes emerged from students’ reflection, cultural immersion worksheets and semi-structured interview schedules. The major themes identified are tagged *the experience; exploring Health and Wellbeing: the lens of host families and recommendations.*

### 3.1. The Experience

The cultural immersion program, as communicated by students, host families and facilitators, was beneficial. Four students who participated felt the experience was significant for them to be able to provide culturally competent and safe healthcare services in their future roles as health professionals. Two students highlighted how valuable the program was:

*This experience has started to open my eyes and understanding of the different stories and journeys people have in seeking to live in Australia. I had not been aware of the difference in experience for coming in on many different visa types.*

(Juliet, social work student)

*I love how this program was not rigid and not following a strict structure. This allowed [for conversations [to be] steered in any direction we deemed correct for each situation. This really helped us gain a more In-depth and multi-faceted experience.*

(Jessica, nursing student)

The facilitators who were the connectors between the students and the host families indicated that the program was incredible and a motivational opportunity to inspire one another.

*The exchange of cultural values and belief between clients and students were incredible. The experience of the clients and also the students in their initial settlement journey in Australia was also a key learning point, with both being able to draw inspiration from one another.*

(Parto, a facilitator)

Further, the host families had a positive impact from the students in the cultural immersion program. A facilitator Parto mentioned that *“[one of] the host family were very proud to speak to people from other cultures, improving their English [language] and learning about their culture”.*

Bahaa (a facilitator) mentioned this as well, stating *“the host family really enjoyed the day…and having [the students] over their house was a unique and refreshing experience”* while another facilitator (Ezra indicated that *[the Syrian] family talked about Syrian contribution to the world historically and how that makes them feel proud.*

### 3.2. Learning Experience

This program provided a medium for students to obtain a deeper understanding of the experiences that impact the wellbeing of the host families, which aligns with the aim of this program, which is to provide cultural encounters that allow for culturally competent and safe engagements with CALD people in the healthcare setting. This broadens their horizon of the families’ lived experiences, transition from their home countries to Australia, access to healthcare and basic amenities, their values, identities, religion and cultural norms and how this all intersects with their health. One of the facilitators highlighted the interaction between a host family and students assigned to that family.

*The students were briefed about the unique experience of the [host] family, where they came from in Afghanistan, their experience and treatment under the Taliban and also the Initial tests/challenges they had faced while in Australia.*

(Bahaa, a facilitator)

One of the students (Juliet) who participated in the program said *“being welcomed into people’s homes deepens the experience and truly helps to increase cultural competence”.*

The program allowed students to see those challenges and obtain first-hand information on the trials faced by the host families within the systems of healthcare delivery and services. The challenges mostly highlighted by families and buttressed by students were language barriers and access to services such as interpreters.

*The challenges I am seeing [includes] accessing better health [and] accessing better services…for me, I see language [as] the major issue. Because if you can’t talk or you can’t get the message across to someone about your health, I don’t know how they can get the required help that they need easily. And if they have to be on a waitlist to see an interpreter, that is going to be a big issue and a big risk to their health as well.*

(Tolani, social work student)

Another student highlights the challenges faced by a member of their host family.

*The main cultural issues that I gleaned, was communication. [The family member] English was of good standard from my perspective and yet still felt confused and in the dark when it came to navigating resources that were created for people like [their family]…[The family member] often felt like [they were] given overcomplicated pathways and resources. [And] would try and communicate that but still felt misunderstood and unhappy with the results.*

(Jessica, nursing student).

This was an issue that was highlighted by all host families and one of the host family recommended a way out: *[there] is the need to have more health information written in* [their] *language and more interpreting services.* (Reported by Derek and Irene, social work students)

While the students saw some challenges faced by the host family, they also saw some positives in their learning experience when it came to food and nutrition. One of the facilitators mentioned:

*[The] families were very much involved in curries and not eating meat on special days. There are lots of chilli and herbs in their curry and felt whether the students liked it or not. However, the [cultural immersion students] enjoyed their curries and very much learned about what to do in caring for them when they are sick or in hospital.*

(Canta, a facilitator).

### 3.3. Exploring Health and Wellbeing: The Lens of Host Family

Students were allowed to ask questions about the host families understanding of health and its importance within their day-to-day lives and culture.

It was stated by Shiv and Mata on their cultural immersion worksheet (social work and nursing students) that their host family defined health as: “*stable and settled…* [as without] *health, you do not have life*” this family also linked health to language and community: “[speaking] *the same language [within the community] gives energy* [and promote their health through a sense of] *connectedness*”.

Another host family spoke about how their religion influences their health and defined what health and wellbeing meant to them as reported by Juliet, a social work student:

*[going to the temple every Friday… and celebrating special days at the temple [supports their health]* and they defined health as *“[eating] healthy Sri Lakan food, [using] a lot of fresh vegetables, regularly [buying] fresh food, use of fire [not] gas [cooker], use of clay pots for cooking and no car, walk[ing] or rid[ing] a bicycle.*

Shiv and Mata also reported that their assigned host family reported that culture impacts overall health outcomes.

*Culture plays an important role in supporting the overall health, especially our mental health. It gives you a sense of direction on what is wrong and right in almost every part of your life.*

### 3.4. Understanding the Nuances between Customs and Traditions

The families shared differences in their ways of life, such as language, food, values, morals, norms, traditions and how they differ from that of Australia, with concluding remarks that they are now learning to adapt to the new ways of Australia.

*One of the host family states that: In Australia, we just put young children [on] our shoulders [but this] is something never do in the first three (3) months of their children’s lives.*

(Reported by Juliet, a social work student)

The quote above gave a sense of understanding of the host family values and culture to the student in their future practice as healthcare providers in the community. One of the students stated.

*So, if I’m in a situation [providing care to persons from their culture] and I need to hold a baby, I just need to be aware of that [beliefs] -not putting the baby on the shoulders.*

(Juliet, social work student)

Another student supported this with their experience from their host family:

*Learning about different cultures can help you provide better person-centred care to patients in the nursing field. I have learnt that Sri Lanka Tamil person may request a shaman (prophet) at the hospital. If this situation arises, I would not be surprised but will be more understanding.*

(Cheryl, a nursing student)

### 3.5. Support from Community Services

A host family also spoke on the support they received from the immediate community in Western Sydney regarding their health and wellbeing *“As a refugee with Physical challenges [arm cut off during the war] and coming from a war country, we are provided with services to cope with our mental health. We have counsellors readily available to consult relating to our PTSD (Post-traumatic stress disorder) …We also have social workers that help us to integrate in the community*” (Reported by Derrick and Irene, social works students)

A facilitator told the students about the support provided by the migrant resource centre to families from CALD groups.

*Group services…Children homework support group–learning for the whole family, yoga and health exercise like golf.*

(Canta, a facilitator)

### 3.6. Recommendation

Recommendations were given by students, cultural immersion facilitators and the host family as well. These were some recommendations pertaining to time allocated to the cultural immersion day. Two facilitators stated:

*It was not enough time for both students and families to engage and share their own culture as well as learn more about their culture.*

(Bahaa)

*The day was short, and the family wished there was more time to talk and enjoy the company of the students.*

(Ezra)

This was exhibited at the end of the cultural immersion with the host families.

*When the time [came] to finish the session, there was a keen interest on both sides when they are going to meet again. That moment was incredible to say goodbye to each other.*

(Bahaa, a facilitator)

Further, students made recommendations towards the future of the cultural immersion program, as a lot was learnt from the it.

*I would highly recommend that this program be introduced into Curriculum, especially for Nursing and Midwifery [program]. In just one day, I have learnt so much*.

(Cheryl, a nursing student)

Another student found the program relevant even in the short time *“they were so informative, I learnt so much from the [families].”* (Juliet, a social work student)

Students indicated the need to visit client outside of their home setting to see the facilitators and barriers that may impact their ability to access services.

*I would suggest that this day be continued further, perhaps in a different setting…to gain even more insight [to the family] experience.*

(Irene, a social work student)

Overall, it was a great experience which was captured in the quotes of one the of facilitators.

*It was a great experience for me and the [families]. The [families] got the opportunity to share part of their culture, religious [beliefs] with students [who also] learn[ed] about their cultures. They appreciated the willingness and respect of the students.*

(Parto, a facilitator)

## 4. Discussion

This study captures the transformational learning experience of students at WSU who engaged in the cultural immersion program in order to give insight on their experience and impact their cultural sensitivity, awareness and competency. As earlier established in the literature, immersion programs promote self-reflection on attitudes towards cultural differences and provide opportunities for students to develop relationships and work with community members, and to apply knowledge and skills acquired during training programs in a supervised practice setting [48]. The thematic analysis of the data shows that the cultural immersion program provided an avenue that can begin the process of learning cultural competency and cultural sensitivity, which shows a shift from an ethnocentric to relativism mindset [49]. This also collaborates the assumptions of Mezirow’s transformative learning theory, that claims that exposing a person to a disorienting situation triggers a reflection process that leads to the reassessment of assumptions and perspectives [38].

As previously mentioned, students were engaged in a one-day cultural immersive experience in the home setting of a host family, which allowed students to ask questions about the host family’s view of health and its importance in their daily lives and culture Students obtained an understanding of barriers and facilitators that impacted their host families’ health in Australia. The students in this study found the cultural immersion program eye-opening, with a clear and meaningful understanding of CALD people’s experience of living in Australia, specific to the Western Sydney Community. This immersive experience enhances students’ awareness and knowledge of other cultural groups outside of their own [50], which involves learning about their cultural values, rituals, expectations, and worldviews in order to provide the groundwork for working effectively and competently with members of other communities [51]. Students who were engaged with host families understood and acknowledged the intersectionality (such as, language, migration, cultural norms, religion and health systems) between their health and wellbeing. The study by Cheon et al. [52] highlights that individuals with different intersections of many identities may have substantially different experiences and meanings of advantages and disadvantages than those who do not. This was a key concept for the students in their immersion experience because they were able to identify that even with the host families being from a CALD background, their experiences were different. In turn, this demonstrates the importance of what being a future healthcare practitioner means and, the need to put these intersections into consideration, and ensuring inclusive [53] and tailored care that is safe for the individual.

The cultural facilitators present in the immersion program served as connectors between the students and their host families. Students received information about their assigned families from their cultural facilitators, making it a comfortable experience for both students and host families. In the research of Smith et al. [10], which focused on the immersion of medical students to the culture of Aboriginal and Torres Strait Islanders, they stated that the students strongly agreed that the facilitators in the program contributed positively to their learning. Onosu [50] indicated in her study of immersion through the study abroad program that participant indicated that the support received from family, friends, program facilitators, and mentors helped them manage the anxieties associated with the cultural immersion experience. The one-day immersion program for students was insightful as they obtained first-hand experiences from these families. However, the cultural facilitators did indicate that more time was needed for students to go in-depth into the lives of the families outside of the home setting, in order to observe where the gaps in their health and wellbeing are most felt in the community. According to Smith and Macintosh [54], the length of time of the immersion experience can also affect cultural competency development; this is not to say that a one-day cultural immersive program will not enable such development, but it may impact the proficiency level, as students may not be exposed to different influences that may impact the health of the target population

Contrary to this thought, Philips et al. [55] indicated in their research that there was no agreement on the optimum approach or requirements to prepare for an immersion experience, nor did there appear to be a conventional timeframe for preparing for an immersion experience. Hoffart et al. [56] stated that the duration of time might not impact cultural competency development, but a time is needed for the student to reflect on their experiences to solidify their gains in cultural competency, which was achieved after the cultural immersion program for students who engaged with host families in this study. Further, there is a unique learning experience that this study has identified. While the study focuses on the immersion of health students, the immersion program also influenced the host families. Host families were able to learn about Australian culture and other cultures that the assigned students identify. This immersion experience also allowed the families to improve their English language, draw inspiration, and exchange meaningful information.

This study shows us from the students’ narrative there is a possibility of a long-term impact of their experience as future health practitioners. The study of Hutchins et al. [57] reported that recent graduates acknowledged that immersion experiences made a difference in their lives both professionally and personally. The evidence shown in the study of Smith and Macintosh [54] indicates that immersion experiences increased student empathy and allowed students to discover respect for differences in language and culture, and over time the experience continued to impact and affect nursing care. Immersion programs demonstrate that after the experience, students may consciously or unconsciously continue to unpack cultural competence constructs [33] and gradually unravel the cultural awareness construct, allowing for self-reflection of personal biases and stereotypes towards culture and religion. Overall, the students who engaged in the program valued the experience, and as Ulvund and Mordal [58] reported in their study, this allowed students to feel more comfortable interacting with people from diverse cultural backgrounds. The value of this experience prompted students to recommend a continuous immersion experience for future students in the health field to engage in the cultural immersion program and for it to be introduced into the curriculum. As stated in the study of Smith and Macintosh [54], which focused on Aboriginal people and Torres Strait Islanders, the health framework created in their study identifies cultural immersion as one of the most effective implementation initiatives for introducing cultural awareness education to medical students.

### 4.1. Limitation of Study

Despite the vast and robust findings and literature related to this research, there are some limitations identified. Firstly, the host families were identified from CALD groups only. However, there is an ever-growing need to take into consideration inclusion of other diverse groups within the Western Sydney region. Another limitation was that only students from health-related disciplines were included in the study. Our future research would explore the cultural immersion experience of any student, irrespective of their discipline, in order for them to engage with diverse groups, including CALD groups. Our subsequent research seeks to address the current gaps in the existing literature to expand the pedagogy of cultural competency outside the scope of health and into other disciplines.

### 4.2. Future Direction

The research team of this study looks to continue studying cultural encounter immersion with a diverse group of students who are not only in the health-related field but students who are passionate in providing services that are culturally competent. Additionally, engagement with diverse communities outside the CALD communities will be explored in Australia, such as diverse religious identities, sexual identities (LGBTQI), persons living with a disability, and the elderly, as well as engagement with the First nations people of Australia. The immersion day will also be lengthened, as recommend by both students and facilitators in this pilot study, to enable students gain insights on how these population groups interact with the community and health services. Currently, the research team are exploring funding opportunities to enable a more robust and large-scale study.

## 5. Conclusions

Evaluation of both the results and existing literature has shown that the cultural immersion program is a highly beneficial approach to orient students early about diverse groups, their culture, and their health. The program encourages students to explore, understand and reflect on their position in health and healthcare in a culturally safe environment through reflexive practices. Students and families demonstrate that this was a positive experience and an enriching one that encouraged the participants to stop and think before they act. The promotion of this invaluable approach ensures that students take these lessons into their practice. This immersion experience has also provided students with an opportunity to learn about their preconceptions of culture, the social constructions of culture, and the cultures of people around them, to provide culturally competent and safe healthcare services. Hence, this valuable and eye-opening experience needs to be continuous to allow our society and health systems to operate through a relativist lens.

## Data Availability

The data are not publicly available; however, they can be provided on request.

## Supplementary Materials

The following supporting information can be downloaded at: https://www.mdpi.com/article/10.3390/ijerph19095114/s1, Table S1: Inclusion and Exclusion criteria.
