# Supplementary material for: From the World to Western: A Community-Engaged Teaching Strategy to Enhance Students’ Learning of Cultural Issues Relevant to Healthcare
Source: Int J Environ Res Public Health. 2022 Apr 22;19(9):5114. doi: 10.3390/ijerph19095114 (PMC9105553; doi:10.3390/ijerph19095114)
Supplement: Supplementary file 1 [file ijerph-19-05114-s001.zip › ijerph-1668905-supplementary.pdf]

Table S1: Inclusion and Exclusion Criteria

	Student	Host Family	Facilitator
**Characteristics of Participants**	Western Sydney University Students who were enrolled in health-related disciplines such as Nursing, Medicine, and Allied Health.	Families that were from culturally and linguistically diverse background from Western Sydney region and were willing to share their history, health practices and beliefs with students from other cultural backgrounds different from theirs. A member of the family was the direct contact person that provided consent and that the students was allocated to work closely with on their respective days of cultural immersion.	Case officers/ workers who work with host families in Western Sydney region and were willing to participant in the cultural immersion program.
**Inclusion criteria**	To participate in the program, students must meet the inclusion criteria of (1) a student of Western Sydney University and enrolled in a health related course/discipline (2) willingness to reflect on their cultural background and willing to share their perspectives on their history, health practices and beliefs (3) embrace diversity and willing to know more about other cultures, history, health practices and beliefs (4) willing to spend a day with a family from a cultural background that is different from theirs (5) willing to be paired with another student from a discipline and cultural background that is different from theirs (6) socially intelligent (7) 18 years and over (8) available during in the months of January and February of 2021.	(1) Families of CALD background (migrated to Australia as a migrant or refugee) (2) hospitable (3) Have a conversational command of English language (4) proud of their cultural background and willing to share their history, health practices and beliefs (5) embrace diversity and willing to know more about other cultures (6) willing to spend a day with 2 undergraduate students from another cultural background different from theirs (7) 18 years and over (8) available during the month of January and February 2021.	Case officers or workers from the Community groups, migrant resource centres and non-government organisations that provide services to CALD families in Western Sydney region.
**Exclusion criteria**	(1) Western Sydney University students who are not enrolled in health-related disciplines (2) Less than 18 years of age (3) Lack of willingness to reflect on and share their perspectives on their history, health practices and beliefs (4) Lack of willingness to spend a day with a family from another cultural background different from theirs (5) non-availability during the designated months	(1) Inability to engage in conversational English (2) Less than 18 years of age (3) Lack of willingness to share their history, health practices and beliefs (4) Lack of willingness to spend a day with two undergraduate students from another cultural background different from theirs (5) non-availability during designated months.	Case officers or workers who are not from the Community groups, migrant resource centres and non-government organisations that provide services to CALD families in Western Sydney region.
